# Ciliopathies - from rare inherited cystic kidney diseases to basic cellular function

**DOI:** 10.1186/s40348-015-0019-1

**Published:** 2015-05-19

**Authors:** Sandra Habbig, Max Christoph Liebau

**Affiliations:** Department of Pediatrics and Center for Molecular Medicine, University Hospital of Cologne, Kerpener Straße 62, 50937 Cologne, Germany; Department of Medicine II, Nephrology Research Laboratory University Hospital of Cologne, Kerpener Straße 62, 50937 Cologne, Germany

**Keywords:** Cystic kidney disease, Cilia, Ciliopathy, Rare Genetic Diseases, PKD, Nephronophthisis

## Abstract

**Background:**

Primary cilia are membrane-bound microtubule-based protuberances of the cell membrane projecting to the extracellular environment. While little attention was paid to this subcellular structure over a long time, recent research has highlighted multiple cellular functions of primary cilia and has brought cilia to the focus of medical and cell biological research.

**Findings:**

Cilia are nowadays considered to be crucial cellular structures controlling diverse intracellular signaling cascades. Dysfunction of cilia leads to a pleiotropic group of diseases ranging from cystic kidney disease via neurologic disorders to metabolic phenotypes and cardiac malformations. According to the underlying cellular pathophysiology, these diverse disorders have been subsumed under the term “ciliopathies”.

**Conclusions:**

The work on rare human ciliopathies has strongly deepened our genetic and cell biological understanding of multiple diseases and cellular events thus ultimately leading to clinical trials of novel therapeutic approaches. This review focuses on some of the important developments in ciliopathy research.

## Polycystic kidney diseases paved the way for establishing the concept of ciliopathies

**Figure 1:**
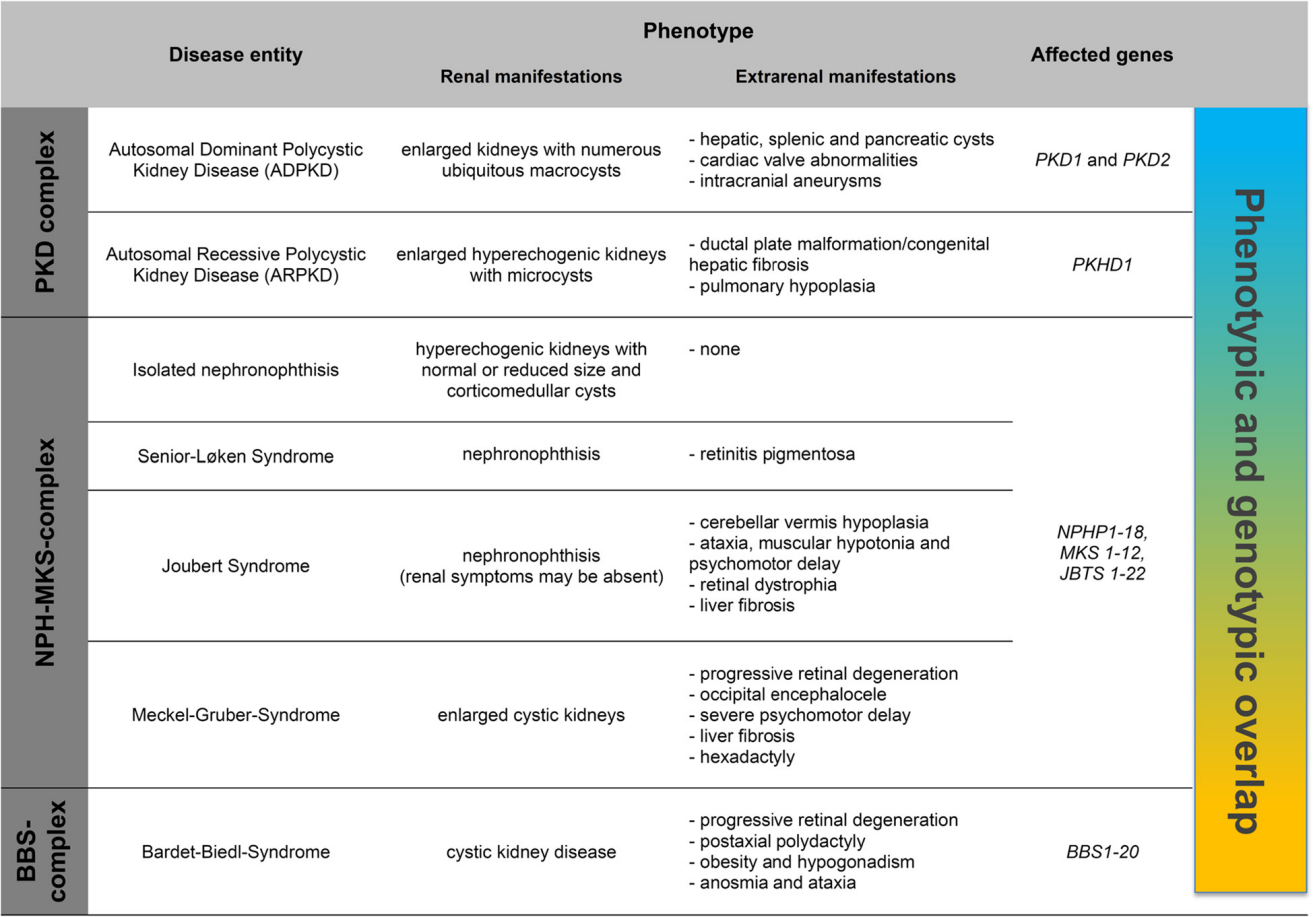
Clinical synopsis of the main disease entities and overview over the affected genes.

**Figure 2:**
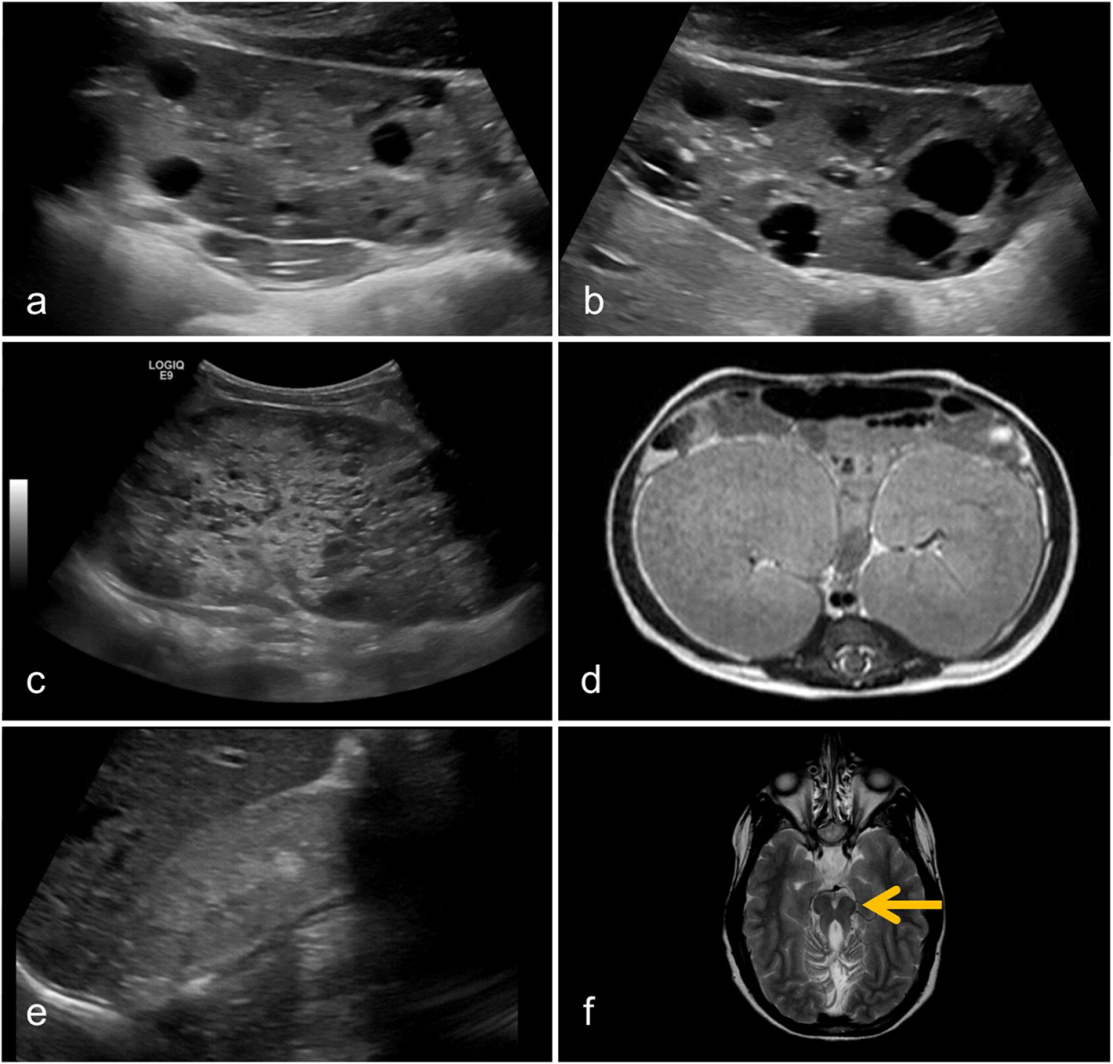
Typical radiological findings in children with cystic kidney disease. **(a, b)** Typical ubiquitous macrocysts and enlarged kidney volumes are found a 15-year-old boy with ADPKD. **(c)** ARPKD typically presents with hyperechogenic kidney with microcysts as shown in a sonography of a 1-year-old boy. **(d)** The massively enlarged kidney volume in ARPKD is illustrated on axial abdominal MRI of a 10-month-old girl. **(e)** Ultrasonography of patients with nephronophthisis often shows small, hyperechogenic kidneys without corticomedullar differentiation. If present, cysts are typically found at the corticomedullar border. **(f)** Cerebellar vermis asplasia and elongated superior cerebellar peduncles result in the *Molar Tooth Sign* on axial MRI, which is pathognomonic for Joubert syndrome. ADPKD, autosomal dominant polycystic kidney disease; ARPKD, autosomal recessive polycystic kidney disease.

Understanding the pathophysiological events underlying rare genetic disorders has been a challenging task over a very long time, until the genetic revolution and novel techniques allowed high-throughput study approaches. As an example, the genetics of the mostly rare pediatric cystic kidney diseases remained very poorly understood. These disorders show a high degree of genotypic and phenotypic variability. Cystic kidney disease may present before birth like autosomal recessive polycystic kidney disease (ARPKD), during childhood and adolescence like nephronophthisis, or mostly in adults like the autosomal dominant polycystic kidney disease (ADPKD) which is one of the most frequent monogenetic diseases with an incidence of 1:1,000. Ciliopathies may show an isolated renal phenotype or may form part of a wide range of syndromes with partly overlapping renal and extrarenal clinical symptoms [1-4]. As an example, nephronophthisis may be found isolated, combined with retinitis pigmentosa in Senior-Løken syndrome or in a more severe syndrome with additional vermis asplasia in Joubert syndrome. Figure 1 aims to give an overview over the spectrum of diseases and clinical symptoms associated with genetic cystic kidney diseases. As pointed out in more detail below, multiple genes can be affected in disorders displaying cystic kidneys and the interplay of these genes strongly affects a patient’s phenotype. Examples of clinical symptoms are shown in Figure 2. For a more detailed description of clinical features, we refer to some of the excellent reviews on ciliopathies [1,5-7].

It was work on a kidney-free model organism that would open up a novel field of cell biological renal research. In 1999, Maureen Barr’s studies on the nematode *Caenorhabditis elegans* identified a link between the gene product most frequently affected in the adult-onset common form of polycystic kidney disease, autosomal dominant polycystic kidney disease (ADPKD), and primary cilia. Surprisingly, the authors could show that the polycystin-1 homologue in *C. elegans* not only localized to cilia of sensory neurons but was also required for the function of these cells [8]. Only a year later, this first connection between cilia and cystic kidney diseases was strongly supported by the findings of Gregory Pazour and colleagues. By identifying the underlying genetic cause of a murine model of cystic kidney disease, these researchers identified a ciliary transport protein required for ciliogenesis both in mice as for the cilia-like flagellae of the green alga *Chlamydomonas rheinhardtii* [9]. Cilia of tubular cells in the cystic kidneys of affected animals were dramatically shortened [9]. Over the following years, multiple disease genes associated with cystic kidneys were identified, and almost all of the corresponding gene products were found to localize to primary cilia [1,6]. The localization of affected gene products at the cilium in combination with a cystic kidney phenotype after targeted inactivation of essential ciliary genes led to the so-called ciliary hypothesis. According to this hypothesis, cilia regulate intracellular signaling pathways, and a dysregulation of these pathways can result in cystic kidney disease [4].

## Cilia function as sensory organelles of a cell

**Figure 3:**
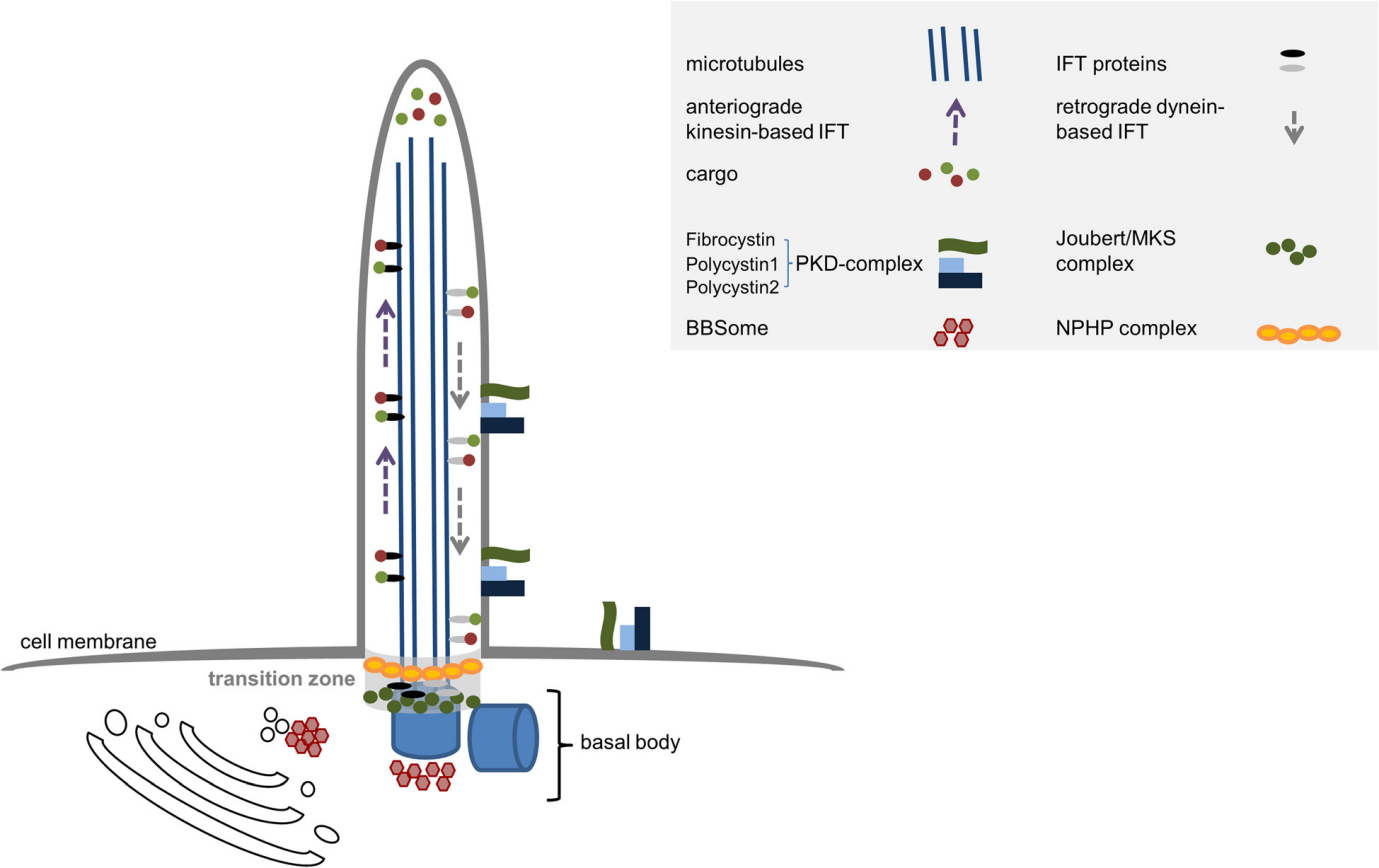
Schematic illustration of cilia and ciliary protein complexes. Gene products affected in different ciliary phenotypes are found in common protein complexes and frequently show functional overlap. Transport into and within a cilium is regulated by kinesin- and dynein-based intraflagellar transport.

So what are primary cilia and what is their function? Primary cilia are small singular cellular organelles that can be found on the cellular surface of nearly every human cell type [1-3]. They consist of a microtubular-based cilioskeleton, the ciliary axoneme, which is surrounded by a specialized cellular membrane. The ciliary axoneme develops on the basis of the more mature of the two centrioles forming a centrosome. During early interphase, this so-called mother centriole gets attached to the cellular membrane and develops into the basal body of a cilium. The microtubular axoneme of primary cilia consists of a ring of nine microtubular doublets, is highly acetylated, and undergoes growth and shrinkage at its distal end [1-3]. As cilia do not contain ribosomes and as protein entry into the ciliary compartment is tightly regulated, transport within a cilium requires a cargo system to deliver substrates to the ciliary tip. This ciliary or “intraflagellar” transport occurs in a kinesin-based fashion on the way to the ciliary tip with microtubular plus-ends and in a dynein-based fashion on the way back to the basal body (see Figure 3). Interference with either transport system can result in ciliary dysfunction and ciliopathy-like phenotypes [1-3].

Despite overwhelming progress over the last 15 years, ciliary function remains incompletely understood. While different subfunctions have recently been described, two main concepts have been suggested for ciliary function. It has been proposed that cilia may have mechanosensory functions sensing flow in the renal tubule and that the two proteins affected in ADPKD may jointly act as mechanosensors. Polycystin-1 is a very large transmembrane protein with a long extracellular part and short cytoplasmic tail, which is involved in the regulation of multiple pathways, some of which are linked to growth control [10]. Polycystin-2 is a nonselective cation channel from the family of transient receptor potential ion channels [10]. Dual photon microscopy could indeed show that cilia in the renal tubule are being bent over *in vivo* [11]. Initial work found a cytosolic polycystin-dependent calcium response in response to flow [12]. However, recent work points to restricted intraciliary rather than cytoplasmic calcium effects subsequently regulating cellular sonic hedgehog signaling [13,14].

A second possible function of cilia is chemosensation. According to this concept, cilia sense the outside of the cell and act as little antennae. Indeed, various cilia-specific receptors have been identified, including receptors for somatostatin and platelet-derived growth factor AA (PDGF AA) [1-3]. Furthermore, crucial effects of cilia and cilia-associated proteins have been described for various important intracellular signaling pathways such as sonic hedgehog signaling or WNT signaling. Cilia-dependent signaling affects very basic cellular functions and aspects including cellular polarity and cell cycle progression [1-3].

In addition to mechano- and chemosensation, there is evidence for further sensory function of cilia, *e.g.*, for photosensation, osmosensation, thermosensation, and olfactory sensation [1]. It was via the knowledge of the underlying cellular pathophysiology that novel clinical phenotypes were recently described, *e.g.*, in Bardet-Biedl syndrome (BBS). Anosmia and defects in peripheral thermosensation and mechanosensation were only detected in BBS patients, when specifically looked for after the identification of BBS as a ciliopathy [15,16].

## Ciliopathies as models for genetic interaction

BBS is also a good example for our current understanding of genetic and functional ciliary cell biology. The BBS phenotype can be caused by a variety of genes. Mutations in 20 genes have currently been described as the cause of BBS [6], which may initially seem to be a surprisingly high number. Still, the observation that a large group of the affected gene products form a joint “BBSome”-protein complex [17] makes it easier to understand that mutations in so many different genes result in the same phenotype. This landmark finding strongly suggests that the overlapping phenotypical and clinical presentation of mutations is directly linked to a common cellular function of the affected gene products within the cell.

According to our current understanding, the phenotype of a ciliopathy patient can therefore be regulated by the genotype in multiple ways. While it is obviously important which gene is affected by a mutation, the phenotype may also change according to the type of mutation, *i.e.*, missense mutation vs. truncating mutation. A good example are the different subtypes of mutations in the *CEP290* gene, which can either result in isolated nephronophthisis, in the more severe Joubert syndrome, or in the most severe, often embryonic lethal Meckel-Gruber syndrome [6]. Comparable reports have been described for other ciliopathy genes. For *NPHP3*, the phenotypic variability can be explained by the type of mutation with biallelic missense mutations resulting in the less severe phenotype of isolated nephronophthisis and two truncating mutations resulting in a Meckel-Gruber phenotype [18]. In addition to the type of mutation and the affected gene, the concept of “mutational load” with evidence of genetic interaction of different ciliopathy genes, has received much attention [1,6]. Various cilia-associated disorders show more severe phenotypes in patients with additional modifying mutations in functionally related ciliary genes as seen in patients with homozygous *NPHP1* deletions, in whom, *e.g.*, additional *NPHP6* mutations could be detected in patients with a more severe neurological phenotype [19]. Finally, some genetic alterations *per se* may also not lead to the clinical phenotype but only become clinically relevant in patients with other mutations in ciliary genes. Combined heterozygous mutations in two autosomal recessive genes might therefore result in truly “oligogenic” inheritance [1,2,6], as suggested for Bardet-Biedl syndrome [20].

Overall, a concept emerges according to which the amount and severity of mutations in functionally related genes are crucial for the clinical phenotype. As different ciliopathy protein complexes can be found in different subcompartments of this cellular organelle, such a concept includes the possibility of differential phenotypes according to the different affected subparts of the cilium.

## Future directions: where do we go from here?

Even though we have learned a lot about cilia, cilia-associated genetics and ciliary signaling major questions remain open. What are the exact cellular functions of the proteins affected in PKD and how do they affect each other? What is the relationship between cilia and other cellular organelles? Can we identify altered signaling pathways in PKD that can successfully be modified by pharmacological interference in patients? These are only a few examples of questions that need to be addressed. Our incomplete understanding of the molecular was recently illustrated by a very elegant study on a mouse model of ADPKD. In this study, the researchers knocked out the ADPKD genes *Pkd1* or *Pkd2* as well as *Kif3a* or *Ift20*, two proteins essential for ciliogenesis. As the single knockout of either of these genes results in polycystic kidneys, it came as a major surprise that the additional loss of cilia in orthologous ADPKD mouse models dramatically weakened the phenotype [21]. Thus, while loss of cilia, loss of ciliary structure, or loss of ciliary signaling without doubts results in cystic kidney, a ciliary pathway might be overactive and contributing to progressive cyst growth during the course of ADPKD. While detailed studies on cilia-related pathways have revealed numerous pathways regulated by or in the cilium, the interplay of different pathways remains very poorly understood and requires further studies.

Furthermore, the functional relation of cilia with other cellular structures remains poorly understood. Cilia obviously contribute to cellular polarity, but they are also the result of cellular polarity as primary cilia will only form on one side of the cell, *e.g.*, the luminal side of a tubular epithelial cell [2]. The interaction of cilia with tight junctions may become another interesting field of research. Additionally, cilia-related proteins have also been found in extraciliary organelles. A prominent example is the nuclear localization of various ciliopathy proteins that have been shown to regulate gene expression networks [22,23]. Finally, recent work points to a role of the cilium as a sender and not only as a sensor. Cilia may be excreting exosomes contributing to intercellular signaling. Exosomes containing PKD-proteins have been described in human urine, but their function remains to be determined [24].

Clinically, the field of targeted therapeutic approaches for patients with rare diseases is just emerging. As described above, rare genetic disorders can teach us a lot about basic pathophysiological principles, and we may in the future be able to apply these principles to other, more common, disorders. As an example, ciliopathies like BBS are associated with diabetes mellitus and obesity, and mouse models suggest a role for cilia in controlling hyperphagia [25]. Different cilia-associated signaling pathways are closely linked to tumorigenesis and ciliary signaling affects the course of, *e.g.*, in mouse models of medulloblastoma [26,27]. Most recent work supports a role for cilia and cilia-associated signaling in the pathogenesis of cardiac malformations [28]. Therefore, the link of cilia to more common disorders seems very plausible and will be followed-up over the next years.

In summary, cilia have become a central topic in cellular research. The novel cellular insights have led to the establishment of a novel molecular understanding in pediatric cystic kidney diseases and various clinical trials, again underlining the importance of basic science for the progress in disease-oriented research [29].
